# Delays in Time-To-Antibiotics for Young Febrile Infants With Serious Bacterial Infections: A Prospective Single-Center Study

**DOI:** 10.3389/fped.2022.873043

**Published:** 2022-04-29

**Authors:** Jinghui Yang, Wei Jie Ong, Rupini Piragasam, John Carson Allen, Jan Hau Lee, Shu-Ling Chong

**Affiliations:** ^1^Department of Paediatric Medicine, KK Women's and Children's Hospital, Singapore, Singapore; ^2^Duke-NUS Medical School, Singapore, Singapore; ^3^Department of Emergency Medicine, KK Women's and Children's Hospital, Singapore, Singapore; ^4^Centre for Quantitative Medicine, Duke-NUS Medical School, Singapore, Singapore; ^5^Department of Paediatric Subspecialties, Children's Intensive Care, KK Women's and Children's Hospital, Singapore, Singapore

**Keywords:** serious bacterial infections, infants, recognition delay, sepsis, antimicrobial therapy

## Abstract

**Introduction:**

Fear of missed serious bacterial infections (SBIs) results in many febrile young infants receiving antibiotics. We aimed to compare the time to antibiotics between infants with SBIs and those without.

**Materials and Methods:**

We recruited febrile infants ≤ 90 days old seen in the emergency department (ED) between December 2017 and April 2021. SBI was defined as (1) urinary tract infection, (2) bacteremia or (3) bacterial meningitis. We compared the total time (median with interquartile range, IQR) from ED arrival to infusion of antibiotics, divided into (i) time from triage to decision for antibiotics and (ii) time from decision for antibiotics to administration of antibiotics.

**Results:**

We analyzed 81 and 266 infants with and without SBIs. Median age of those with and without SBIs were 44 (IQR 19–72) and 29 (IQR 7–56) days, respectively (*p* = 0.002). All infants with SBIs and 168/266 (63.2%) infants without SBIs received antibiotics. Among 249 infants who received antibiotics, the median total time from ED arrival to infusion of antibiotics was 277.0 (IQR 236.0–385.0) mins for infants with SBIs and 304.5 (IQR 238.5–404.0) mins for those without (*p* = 0.561). The median time to decision for antibiotics was 156.0 (IQR 115.0–255.0) mins and 144.0 (IQR 105.5–211.0) mins, respectively (*p* = 0.175). Following decision for antibiotics, infants with SBIs received antibiotics much faster compared to those without [107.0 (IQR 83.0–168.0) vs. 141.0 (94.0–209.5) mins, *p* = 0.017].

**Conclusion:**

There was no difference in total time taken to antibiotics between infants with SBIs and without SBIs. Both recognition and administration delays were observed. While all infants with SBIs were adequately treated, more than half of the infants without SBIs received unnecessary antibiotics. This highlights the challenge in managing young febrile infants at initial presentation, and demonstrates the need to examine various aspects of care to improve the overall timeliness to antibiotics.

## Introduction

Serious bacterial infections (SBIs) are a preventable source of infant mortality, with the majority of these deaths attributed to sepsis, meningitis and pneumonia (1–3). Although most of these deaths are in low- to middle-income countries (3), sepsis alone still accounts for an estimated 11% of young infant mortality in high-income countries (4). Survivors develop disabilities such as cerebral palsy, impaired growth, and suffer from cognitive deficits (5–8).

Recognizing that SBIs have potentially devastating consequences, clinicians have developed guidelines for the optimal management of sepsis, pneumonia and urinary tract infection (UTI), where judicious and timely administration of antimicrobial therapy is critical (9–11). The Surviving Sepsis Campaign (SSC) International Guidelines recommend starting antibiotics as soon as possible, within 1 h of recognizing a child with septic shock, or within 3 h of recognizing sepsis-associated organ dysfunction without shock (9). Early administration of antibiotics, has been demonstrated to reduce subsequent resource utilization, including progression to organ dysfunction, hospital length of stay (LOS), and mortality (12, 13).

Young infants <90 days old with SBIs pose a diagnostic challenge to emergency department (ED) physicians and pediatricians. Algorithms such as the Rochester criteria, Boston criteria and the Philadelphia criteria were developed to guide physicians to differentiate between high and low-risk infants (14–16). Newer protocols like the “Step-by-Step” approach and the Pediatric Emergency Care Applied Research Network (PECARN) rule were derived to define low-risk febrile infants and were found to have a higher sensitivity and negative predictive value than the RC (17, 18). Although the newer algorithms focus on defining a low-risk group, these protocols are not used systematically in clinical practice (19–21).

Due to fear of missed SBIs, there is a low threshold to perform invasive investigations and administer empirical antibiotics to most febrile infants. While this ensures that clinicians err on the side of caution, the suboptimal specificities (46.9–60.0%) of the most recent risk stratification tools mean that febrile infants without SBIs are also extensively investigated and treated (17, 18). This may result in delays in antibiotic administration, especially considering the busy nature of the emergency department, where overcrowding and resource management can challenge the provision of timely optimal care.

Delays in time-to-antibiotics are divided into 2 categories: First, time from arrival in the ED to time of decision to administer antibiotics, which requires prompt recognition of a child with SBI and measures “recognition delay”. Second, time from decision to administer antibiotics to time of actual administration of first dose antibiotics, which measures “administration delay” in the delivery of antibiotics (22). Both delays are associated with increased mortality (22–24). Studying these 2 key time points would allow targeted interventions to improve clinical outcomes.

Our primary aim was to compare the timing of antibiotic administration between young infants with SBIs and those without, divided into: (i) total time from ED arrival to infusion of antibiotics, (ii) time from arrival in the ED to time of decision to administer antibiotics (“recognition delay”), and (iii) time from decision to administer antibiotics to time of first dose antibiotics administered (“administration delay”). We hypothesized that young infants with SBIs would have an overall shorter time to antibiotics and have both shorter recognition and administration delays, compared to infants without SBIs.

## Article Types

Tier 1 article: Original Research.

## Materials and Methods

### Study Design, Setting and Population

**Figure 1:**
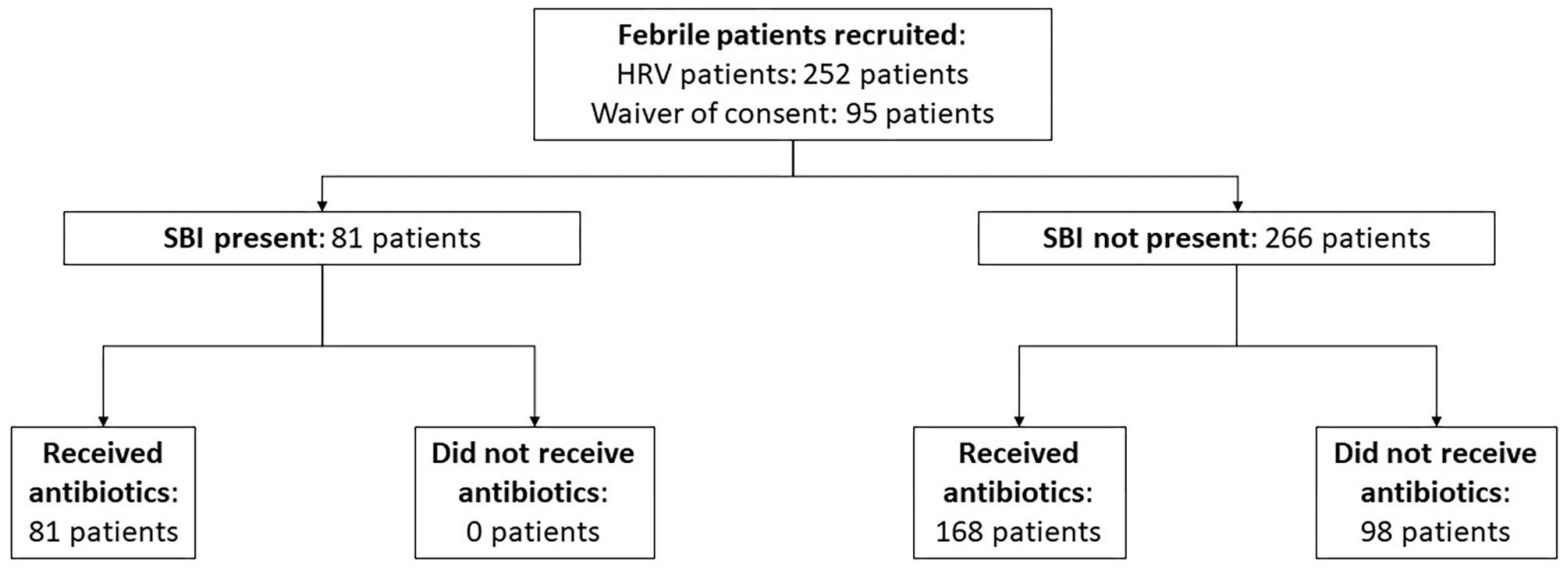
Flowchart of patient recruitment.

We performed a prospective cohort study among infants ≤ 90 days old, with fever, who attended the ED of KK Women's and Children's Hospital (KKH), Singapore between December 2017 and April 2021. The initial cohort was a secondary analysis of infants recruited for a heart rate variability study, from December 2017 to December 2020 (25). Subsequently, we obtained ethics approval to study all febrile infants from January to April 2021 (Figure 1). KKH is one of the two major tertiary centers for pediatric care in Singapore.

We excluded premature infants with a gestation of <35weeks, infants with major congenital malformations, chronic respiratory conditions requiring home non-invasive support or perinatal conditions requiring at least 7 days of stay in the neonatal intensive care unit prior to this hospitalization. We chose to exclude these because they formed a high-risk population that would automatically receive urgent attention in the ED and inpatient services and the approach is different from other febrile infants. We also excluded infants who received antibiotics in the last 48 h before presenting to the ED, and infants who were discharged against medical advice from the ED.

In our institution, infants ≤ 90 days old who are clinically unwell or remain febrile (temperature ≥38.5°C or ≥38.0°C on 2 occasions) will receive a full septic work up including full blood count, C-reactive protein (CRP) and procalcitonin, blood, urine and cerebrospinal fluid (CSF) cultures. They will also be started on empirical intravenous antibiotics while awaiting culture growth. For infants who remain afebrile or do not reach the fever criteria during the hospital stay, a urinalysis is ordered. Subsequent investigations and decision for antibiotics are physician-dependent. The decision for antibiotics in these cases is based on the clinical status as well as initial biochemical results including CRP and procalcitonin. All infants hospitalized for fever are monitored until they are well and afebrile for 24 h before discharge.

### Study Definitions

Fever was defined as an axillary or rectal temperature of ≥38.0°C. SBI was defined as (1) UTI, (2) bacteremia or (3) bacterial meningitis (26). UTI was defined as growth of a single pathogen of (1) >100,000 colony-forming unit (CFU)/ml in a clean catch specimen, or (2) ≥50,000 CFU/ml in a catheterized specimen, or (3) 10,000–50,000 CFU/ml in a catheterized specimen with an abnormal urinalysis (positive for leucocyte esterase, nitrite or >5 white blood cell/per high-powered-field). Bacteremia was defined as growth of a single pathogen in blood culture. When the bacteria was considered to be a likely contaminant, for example *coagulase-negative staphylococcus*, it was excluded. Bacterial meningitis was defined as (1) growth of a single pathogen in the CSF, or (2) sterile CSF pleocytosis with biochemical changes consistent with bacterial meningitis where CSF protein is more than 100 mg/dL and CSF to plasma glucose ratio <0.6 (27, 28). Invasive bacterial infection (IBI) is defined as bacterial meningitis and/or bacteremia (29).

### Data Collection

Data on baseline characteristics was collected prospectively from medical records and entered into a structured data entry form. We collected data on age, sex, presence of co-morbidities, prematurity status and vital signs at the ED.

Laboratory investigations included full blood count, CRP, procalcitonin and blood, urine and CSF cultures.

### Outcome Measures

We computed the time taken to antibiotics, defined as total time from ED triage to the administration of the first dose of antibiotics. The timing was further divided into (i) time taken from arrival in the ED to decision to administer antibiotics (“recognition delay”), and (ii) time between the decision to administer antibiotics to time which the first dose of antibiotics was administered (“administration delay”). Time stamps were collected from the electronic health record by a study team member who was blinded to the SBI status of the study subjects. The recognition delay time stamp was taken as the time the attending physician reviewed the infant and made the decision for antibiotics, as captured by the physician's consult note in the electronic health record. In cases where the attending physician made a note to monitor for a fever spike before starting antibiotics, the time of the fever spike was used as the recognition delay time stamp. We also computed the need for fluid resuscitation, use of inotropes, admission to the high dependency unit (HDU) or intensive care unit (ICU), total hospital LOS, and mortality.

### Statistical Analyses

Categorical data for baseline demographics, clinical characteristics and laboratory results were summarized as frequency counts and percentages and compared between study groups using a chi-square test; continuous data was summarized as median (interquartile range, IQR) and compared between study groups using a Wilcoxon rank-sum test. The Wilcoxon rank sum test was used to compare time to antibiotics between study groups with differences reported using the Hodges-Lehmann shift estimate (median of all possible differences) with 95% confidence interval. Statistical significance was set at *p* ≤ 0.05. We performed a sensitivity analysis defining SBIs as those with culture positive growth, thereby excluding those who were diagnosed with meningitis based on CSF pleocytosis with biochemical changes. We also performed a subgroup analysis on neonates (<28 days old). Analysis was performed using SPSS v26 (Chicago) (30) and SAS v9.4 (SAS Inc. Cary NC) (31).

## Results

### Patient Demographics and Clinical Characteristics

Among 347 patients analyzed, 81 infants (23.3%) had SBIs and 266 infants (76.7%) did not have SBIs. There were 98 neonates, 28 (28.6%) had SBIs and 70 (71.4%) did not have SBIs. Infants with SBIs were older (median 44 days, IQR 19–72 vs. 29 days, IQR 7–56, *p* = 0.002), more likely to be males (80.2% vs. 51.9%, *p* <0.001), had a higher temperature (median 38.5°C, IQR 38.2–39.2 vs. 38.3°C, IQR 38.1–38.7, *p* = 0.013), and had a higher heart rate (median 166/min, IQR 153–181 vs. 160/min, IQR 147–177, *p* = 0.035) than infants without SBIs (Table 1). Among the infants who received blood investigations, infants in the SBI group had higher median inflammatory markers like white blood cell count, absolute neutrophil count, CRP and procalcitonin (Table 2).

**Table 1:** Baseline demographics, clinical characteristics and outcomes of study cohort.

**Variables**	**Patients with SBI (*N =* 81)**	**Patients with no SBI (*N =* 266)**	***P*-value**
Age, days	44 (19–72)	29 (7–56)	0.002
Male gender	65 (80.2%)	138 (51.9%)	<0.001
Co-morbidities	3 (3.7%)	7 (2.6%)	0.614
Prematurity*	6 (7.4%)	14 (5.3%)	0.468
Temperature reading, °C	38.5 (38.2–39.2)	38.3 (38.1–38.7)	0.013
Heart rate per minute	166 (153–181)	160 (147–177)	0.035
Respiratory rate per minute	40 (36–45)	40 (40–45)	0.424
Length of hospital stay, days	4 (3–7)	3 (2–4)	<0.001

*SBI, serious bacterial infection; IQR, inter-quartile range.*

*Continuous variables summarized as median (IQR) and categorical variables as frequency count (%).*

* *Prematurity refers to late preterm babies who were 35–36 weeks of gestation*.

**Table 2:** Laboratory results of study cohort.

**Variables**	**Patients with SBI (*N =* 81)**	**Patients with no SBI (*N =* 266)**	***P*-value**
**Baseline laboratory values**
Hemoglobin (g/dL)	11.10 (10.10–13.50)	12.90 (10.83–17.10)	<0.001
White blood cell count x 10^9^/L	14.07 (10.65–17.69)	11.73 (9.25–13.99)	<0.001
Absolute neutrophil count x 10^9^/L	7.13 (4.58–9.96)	4.51 (2.97–6.23)	<0.001
Platelet count x 10^9^/L	435 (360–539)	404 (330–481)	0.054
C-reactive protein (mg/L)	36.30 (16.15–54.30)	6.00 (2.30–13.33)	<0.001
Procalcitonin (ug/L)	0.83 (0.23–5.12)	0.20 (0.13–0.62)	0.003
**Cultures performed**
Blood cultures performed	80 (98.8%)	171 (64.3%)	<0.001
Urine cultures performed	81 (100.0%)	178 (66.9%)	<0.001
CSF cultures performed	69 (85.2%)	126 (47.4%)	<0.001

*SBI, serious bacterial infection; CSF, cerebrospinal fluid; IQR, inter-quartile range.*

* *Percentages here take the denominator as the total number of patients.*

*Continuous variables summarized as median (IQR) and categorical variables as frequency count (%)*.

Among 81 infants with SBIs, 70 (86.4%) infants were diagnosed with a UTI. Among 15 (4.3% of all febrile infants) infants with IBI, 8 (53.3%) had the diagnosis of meningitis and 9 (60%) had bacteremia (Table 3). Six out of eight (75%) of the infants with CSF culture negative bacterial meningitis were diagnosed by CSF biochemical changes. All of them had their lumbar puncture done prior to receiving antibiotics. All the infants had their CSF sent for culture and filmarray for various viruses and bacteria including *E. coli, Haemophilus influenzae and Streptococcus agalactiae*. Six patients had multiple-source infections (2 had bacteremia and meningitis while 4 had UTI and bacteremia).

**Table 3:** Microbiology results of infants with SBIs in our study cohort.

**Types of SBI**	**Pathogens**	**No. of patients**	**Total No. of Diagnosed patients**
UTI	*Escherichia coli* *Klebsiella pneumoniae* *Citrobacter koseri* *Klebsiella aerogenes* *Staphylococcus aureus* *Enterobacter cloacae* *Enterobacter hormaechei* *Proteus mirabilis* *Serratia marcescens* *Citrobacter youngae*	53 (75.7%) 5 (7.1%) 3 (4.3%) 2 (2.9%) 2 (2.9%) 1 (1.4%) 1 (1.4%) 1 (1.4%) 1 (1.4%) 1 (1.4%)	70
Meningitis	*Escherichia coli* *Streptococcus agalactiae* None isolated	1 (12.5%) 1 (12.5%) 6 (75%)	8
Bacteremia	*Escherichia coli* *Streptococcus agalactiae* *Klebsiella Pneumoniae*	6 (66.7%) 2 (22.2%) 1 (11.1%)	9

*SBI, serious bacterial infection; No., number; UTI, urinary tract infection.*

* *Percentages here take the denominator as the total number of patients in each SBI group*.

### Antibiotics Timings

**Figure 2:**
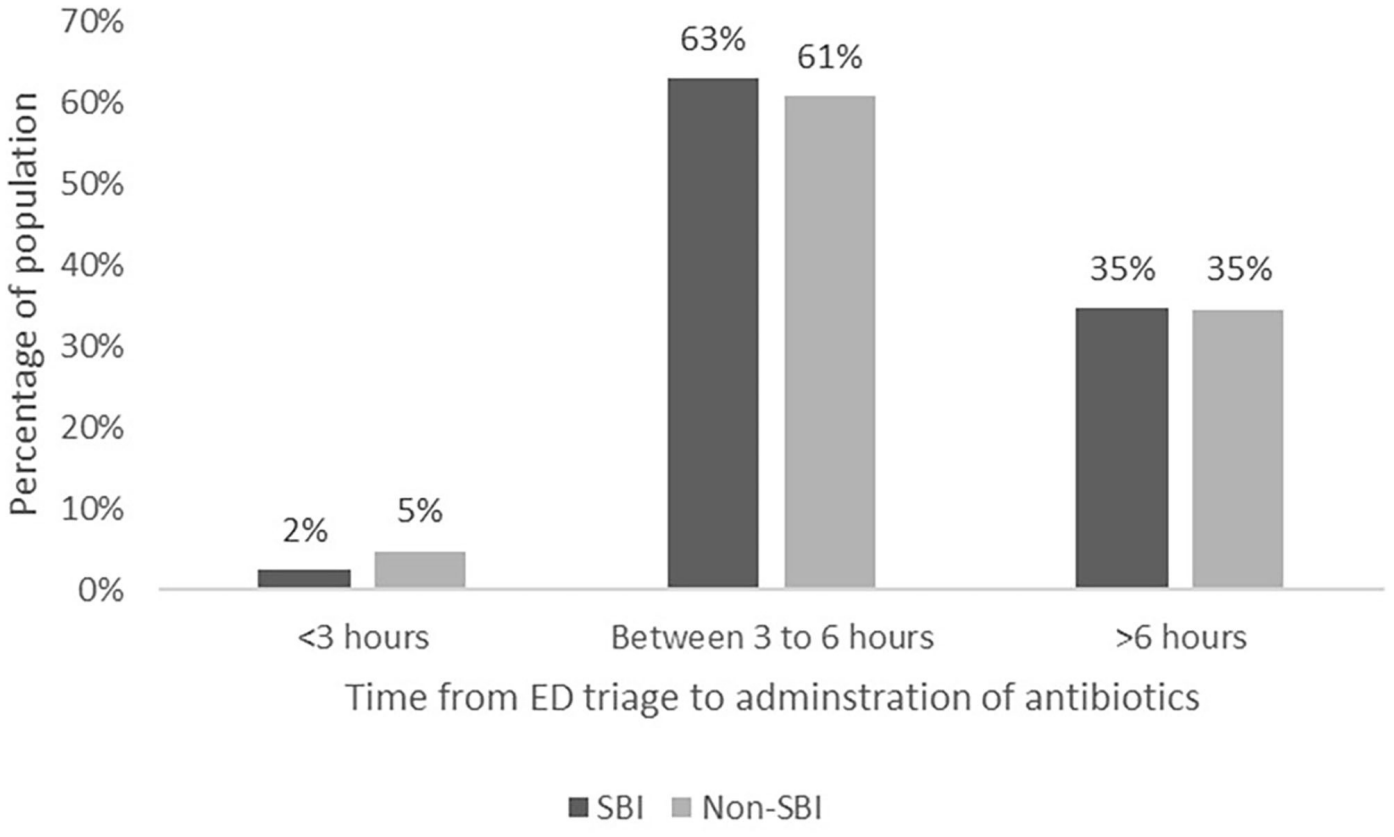
Proportion of infants who received early versus late antibiotics administration.

All infants with SBIs and 168/266 (63.2%) infants without SBIs received antibiotics. As shown in Table 4, the median time from triage at ED to first dose of antibiotics was 277.0 min (IQR 236.0–385.0) in the SBI group, and 304.5 min (IQR 238.5–404.0) in the non-SBI group (difference−8.0 mins, 95% confidence interval (CI)−38.0–21.0, *p* = 0.561). We found no difference in time from arrival in the ED to decision to administer antibiotics in the SBI group (median 156.0 min; IQR, 115.0–255.0) compared to the non-SBI group (144.0 min; IQR, 105.5–211.0) (difference 15.0 mins, 95% CI−7.0–38.0, *p* = 0.175). The median time between the decision for antibiotics and administration of first dose of antibiotics was shorter for the SBI group (107.0 min; IQR 83.0–168.0), compared to the non-SBI group (141.0 min; IQR 94.0–209.5) (difference−24.0 mins, 95% CI−44.0–4.0, *p* = 0.017). Subgroup analysis for neonates (<28 days) yielded consistent results. Figure 2 shows the proportion of infants in each group who received early (within 3 h of presentation) versus late antibiotics administration, as per the SSC guidelines (9).

**Table 4:** Median times related to antibiotics administration for those who received antibiotics.

	**Infants with SBI**,	**Infants without SBI**,		
	**Median (IQR)**	**Median (IQR)**		
**Antibiotic Related Times**	**(*N =* 81)**	**(*N =* 168)**	H-L Shift Estimate (95% CI)*	***P*-value**
Total time taken from ED triage to infusion of antibiotics (minutes)	277.0 (236.0–385.0)	304.5 (238.5–404.0)	−8.0 (−38.0,21.0)	0.561
Time taken from ED triage to decision for antibiotics (minutes) [Recognition delay]	156.0 (115.0–255.0)	144.0 (105.5–211.0)	15.0 (-7.0,38.0)	0.175
Time taken from decision for antibiotics to first infusion of antibiotics (minutes) [Administration delay]	107.0 (83.0–168.0)	141.0 (94.0–209.5)	−24.0 (−44.0, −4.0)	0.017

* *Reference taken as infants without SBIs.*

*SBI, serious bacterial infection; IQR, inter-quartile range; H-L, Hodges-Lehmann; CI, confidence interval; ED, emergency department*.

We performed a sensitivity analysis by excluding cases of CSF pleocytosis without culture positive growth, and found consistent results (Supplementary Table 1). For the outcome of total time taken from ED arrival to antibiotics, there remained no difference between the groups (difference−12.0 mins, 95%CI−60.2–36.2, *p* = 0.625). For time from ED triage to decision for antibiotics, there remained no difference between the groups (difference 13.0 mins, 95%CI−14.0–40.0, *p* = 0.343). For time taken from decision for antibiotics to first infusion of antibiotics, the difference remained significant between the groups (difference−30.0 mins, 95% CI−54.8 to −5.2, *p* = 0.018).

### Secondary Outcomes

Eleven (13.6%) infants with SBIs, and 15 (5.6%) without SBIs received a fluid bolus (*p* = 0.027). Only 1 infant was admitted to the HDU, and 1 other infant to ICU. Both infants had SBIs. There was neither use of inotropes, nor deaths in the study cohort.

## Discussion

In our study of 249 febrile infants who received antibiotics, there was no significant difference in either the overall time taken from triage at ED to first dose of antibiotics, or in the time taken from arrival in the ED to time of decision to administer antibiotics (“recognition delay”) between the SBI and non-SBI cohort. We did find that the SBI group received antibiotics faster than the non-SBI group, once the decision for antibiotics had been made (“administration delay”).

We report a high prevalence of SBIs (23.3%) and IBIs (4.3%) in our cohort. This is higher than that reported by other studies, with a SBI prevalence of 5–15% (32) and an IBI prevalence of 2.3–3.3% (12, 33). We recognize important health service differences in our study population. We are the larger of 2 pediatric tertiary centers in the country and receive referrals from primary care centers. Infants who examine well with a clear source of fever may not be referred to our center for further evaluation. This could have accounted for the higher prevalence of SBIs and IBIs compared to the published literature.

ED providers have difficulty discerning which young febrile infants are at high risk of SBIs. This is likely because infants present with a non-specific complaint of fever and no other reliable signs and symptoms (32). Previously, we demonstrated that current triage tools including the National Institute for Health and Care Excellence (NICE) Traffic Light System and the Severity Index Score (SIS) were insufficient for young febrile infants (34). In our earlier studies, high temperature, tachycardia and a low SIS were associated with serious infections (25, 35). In this study, although there were differences in the height of fever and median heart rate at triage, these were clinically small differences that cannot provide discrimination between infants with SBIs and those without. Many febrile infants continue to be hospitalized and undergo invasive testing and empiric antibiotics treatment (36). Our institution is working to implement a modified algorithm based on the “Step-by-Step” approach to streamline the diagnosis and management of these febrile infants. Using point of care tests with faster turnaround time will provide earlier stratification of febrile infants so that those at high risk of SBIs receive antibiotics more promptly, and infants who are low risk will not be subjected to unnecessary invasive tests and antibiotics.

In our study, we observed that all infants with SBIs (100%) received antibiotics while 168/266 (63.2%) of the infants without SBIs received antibiotics. This underscores our findings that the current approach is highly sensitive in the final diagnosis of SBIs. While we did not miss SBIs in our population (all SBIs received antibiotics), our low threshold for intervention resulted in more than half the infants without SBIs receiving antibiotics. As such, merely treating all infants with SBIs with antibiotics is not sufficient, we seek to identify them early so that we can prioritize empirical antibiotics to those who really need them. This will result in more judicious use of invasive investigations and empirical antibiotics. Once identified, the SBI group received antibiotics faster than the non-SBI group (shorter “administration delay”). This is reassuring that once identified, the hospital has the resources and processes in place to administer antibiotics to infants with likely SBIs rapidly. These include a mechanism for early review in the wards if the young infant is deemed to look unwell or have potential for deterioration, in the ED. Additionally, given that the majority of the infants with SBIs had a UTI, once the urinalysis returned as positive, it may have prompted more timely administration of antibiotics.

The median total time taken from presentation at ED to administration of antibiotics exceeded the SSC recommendation of 3 h for both groups of infants (9). It would be important to examine the various clinical, administrative and logistic processes. The current workflow for febrile infants includes an initial assessment at the ED by the ED physician. Unstable infants receive investigations and urgent antibiotics in the ED. Stable infants are transferred to receive care from the inpatient team, before a decision is made for initiation of antibiotics. The various points of assessment could contribute to a delay in time to antibiotics. Besides focusing on early sepsis recognition, systemic changes to reduce delays could include effective communication between the medical, nursing and bed management teams, robust monitoring systems to detect sudden deterioration in infants, and institution protocols that mandate minimum standards for time to antibiotics.

### Limitations and Strengths

We acknowledge the limitations of our study. Patients recruited in the first part of the study were part of another study and therefore could inadvertently have resulted in selection bias. However, once the team received ethics approval, we were able to track and follow outcomes for all febrile infants from the start of 2021. Not all infants without SBIs had a complete workup performed. However, all febrile infants were monitored until they were well and afebrile for 24 h before discharge, to ensure that no SBIs were missed. Our cohort was largely stable with only 2 infants requiring a higher level of care. We recognize that we may have been underpowered to study difference in time to antibiotics. We used the sample standard deviation (study σ = 350) as an estimate of the population standard (σ). Taking a difference between groups of 60 min as clinically relevant, a sample size of 488 patients per arm would be needed in a similar endeavor to ensure adequate power. Our study provides useful information for sample size computations in future related investigations. Being a single center study, we recognize that our findings may not be generalizable to other healthcare settings. Finally, we recognize that given the rapid changes in clinical status for these young infants, any risk stratification tool, even if methodically derived and validated, may not successfully differentiate infants with SBIs from those without, early in their sickness.

We recognize the strengths of this study, which include prospective data collection, independent verification of time stamps and therefore improved veracity of the timings reported in this study, and none of the subjects were lost to follow-up.

## Conclusion

Given that all our infants with SBIs received antibiotics, our practice is overall safe. However, the goal is an improved level of care where early recognition of infants with SBIs translates to shorter time to antibiotics. We found that when all febrile infants compete for the same resources, early antibiotics was not prioritized for infants with SBIs. Future research should investigate how early accurate risk stratification and robust monitoring systems improve early recognition of SBIs and ensure prompt delivery of antibiotics.

## Data Availability Statement

The raw data supporting the conclusions of this article will be made available on reasonable request to the Corresponding Author.

## Ethics Statement

The studies involving human participants were reviewed and approved by Study title: Rapid Triage for Serious Infections in Infants Younger than 3 months using a Novel Heart Rate Variability Tool IRB No: 2017/2680 SingHealth Centralised Institutional Review Board (CIRB). Written informed consent from the participants' legal guardian/next of kin was not required to participate in this study in accordance with the national legislation and the institutional requirements.

## Author Contributions

JY, WO, JL, and S-LC contributed to the conception and design of the study. JY, WO, and RP collected and organized the database. JA and S-LC performed the statistical analysis. JY and WO wrote the first draft of the manuscript. JA, JL, and S-LC made critical revisions to the manuscript. All authors contributed to manuscript revision, read and approved the submitted version.

## Conflict of Interest

The authors declare that the research was conducted in the absence of any commercial or financial relationships that could be construed as a potential conflict of interest.

## Publisher's Note

All claims expressed in this article are solely those of the authors and do not necessarily represent those of their affiliated organizations, or those of the publisher, the editors and the reviewers. Any product that may be evaluated in this article, or claim that may be made by its manufacturer, is not guaranteed or endorsed by the publisher.
