# Supplementary material for: Delays in Time-To-Antibiotics for Young Febrile Infants With Serious Bacterial Infections: A Prospective Single-Center Study
Source: Front Pediatr. 2022 Apr 29;10:873043. doi: 10.3389/fped.2022.873043 (PMC9099243; doi:10.3389/fped.2022.873043)
Supplement: Supplementary file 1 [file Table_1.DOCX]

Supplementary Table 1: Sensitivity Analysis of median times related to antibiotics administration for infants classified according to their culture outcomes

| Antibiotic Related Times | Infants with **SBI, Median (IQR)  (N=81) | Infants without SBI, Median (IQR)  (N=168) | H-L Shift Estimate (95% CI)* | P-value |
| --- | --- | --- | --- | --- |
| Total time taken from ED triage to infusion of antibiotics (minutes) | 292.0  (253.2-339.3) | 304.0  (280.1-323.9) | -12.0  (-60.2, 36.2) | 0.625 |
| Time taken from ED triage to decision for antibiotics (minutes) [Recognition delay] | 156.0  (138.4-188.3) | 143.0  (128.5-159.4) | 13.0  (-14.0, 40.0) | 0.343 |
| Time taken from decision for antibiotics to first infusion of antibiotics (minutes) [Administration delay] | 108.0  (96.4-134.3) | 138.0  (126.5-160.8) | -30.0  (-54.8, -5.2) | 0.018 |

** Reference taken as infants without SBIs.*

***SBI infants are defined as those with a positive blood, urine or cerebrospinal fluid culture.*

SBI: serious bacterial infection; IQR: inter-quartile range; H-L: Hodges-Lehmann; CI: confidence interval; ED: emergency department
